# Characterizing Shared Sensory Priorities and Age-Group Differences for Feng-Flavor Baijiu: A Consumer Survey Using Hierarchical Weighting and Interpretable Machine Learning

**DOI:** 10.3390/foods15162888

**Published:** 2026-08-18

**Authors:** Xin Yuan, Jiang Xie, Xudong Zhang, Yiyu Chen, Keyi Ling, Bofeng Zhong, Luyang Cao, Dongrui Zhao, Wenjing Tian

**Affiliations:** 1 Key Laboratory of Geriatric Nutrition and Health, Ministry of Education, Beijing Technology and Business University, Beijing 100048, China; 2 Institute of Chinese National Alcohols, Beijing Technology and Business University, Beijing 100048, China; 3 Key Laboratory of Brewing Molecular Engineering of China Light Industry, Beijing Technology and Business University, Beijing 100048, China; 4 Department of Food and Bioengineering, Beijing Vocational College of Agriculture, Beijing 102442, China

**Keywords:** Feng-Flavor Baijiu, consumer sensory perception, SHAP, XGBoost

## Abstract

As Feng-flavor Baijiu increasingly shifts toward consumer-oriented product development, clarifying consumers’ shared sensory priorities and age-related differences is essential for aligning its traditional style with evolving market demands. This study collected 1523 questionnaires across China, of which 1357 valid responses were analyzed. A hierarchical sensory system comprising three primary dimensions—aroma, taste, and drinking perception—and 24 secondary attributes was evaluated using hierarchical weighting, a proportional sensory flavor wheel, machine learning, and SHAP interpretation. Drinking perception received the highest weight (35.23%), although it was only slightly higher than aroma (33.54%) and taste (31.22%). Jiuhai aroma, sweetness, comfort, and purity were identified as the leading attributes within their respective dimensions. Machine learning further identified purity, persistence, saltiness, comfort, and harmony as attributes with relatively high contributions to age-group classification. Age-group differences were not concentrated in any single sensory attribute but were reflected in the classification information jointly provided by multiple sensory attributes. Descriptive group comparisons showed decreasing trends in the relative weights of saltiness and sourness across the 18–30-, 31–45-, and ≥46-year groups, although neither difference remained statistically significant after Benjamini–Hochberg correction. Comfort, bitterness, and honey aroma were relatively more prominent in the ≥46-year group, whereas grain aroma was relatively more prominent in the 31–45-year group. Sweetness and Jiuhai aroma had relatively high overall weights but contributed less to age-group classification, suggesting that they may represent more broadly shared sensory priorities across age groups. These findings identify candidate sensory priorities and segmentation indicators for subsequent validation using actual products, consumer liking tests, and ideal-intensity assessments.

## 1. Introduction

Baijiu is a traditional distilled spirit produced primarily from grains through processes including Daqu preparation, solid-state fermentation, distillation, aging, and blending. It serves multiple functions related to food and beverage consumption, cultural heritage, and social interaction [1]. China is a major producer and consumer of Baijiu [2,3]. According to data from the National Bureau of Statistics of China, Baijiu enterprises above the designated size produced 4.145 million kiloliters in 2024, generating sales revenue of RMB 796.38 billion and total profits of RMB 250.87 billion, demonstrating the substantial scale and economic value of the industry. However, with slowing consumption growth and intensifying competition among alcoholic beverage categories, the Baijiu industry is shifting from expansion-driven growth toward competition within a mature market. Accordingly, product development is moving from a production-oriented approach toward consumer-driven quality optimization and sensory-style expression [4,5]. During this transition, the consumer structure of the Baijiu market is becoming increasingly diverse. In particular, younger consumers are showing new patterns in drinking occasions, product exposure, and expectations regarding the quality and consumption experience of alcoholic beverages [6]. These changes suggest that different age groups may not evaluate alcoholic products according to identical sensory criteria, further highlighting the need to identify consumer sensory needs from a demographic segmentation perspective. Therefore, as the Baijiu industry shifts from a production-oriented to a consumer-oriented model, identifying overall consumer preferences alone is no longer sufficient to support differentiated product development; it is also necessary to clarify the shared and heterogeneous characteristics of sensory evaluation structures across age groups. Therefore, establishing a quantitative sensory evaluation system from the consumer perspective and systematically identifying shared demands and group heterogeneity are essential for improving the alignment between Baijiu product development and market needs and for supporting differentiated product design.

The flavor profile of Baijiu is a fundamental determinant of its quality characteristics and product differentiation [7,8], and its sensory evaluation results from the integrated perception of multidimensional information, including aroma, taste, and drinking perception [9,10]. Different sensory signals may also exhibit synergistic, suppressive, and dynamic interactive effects [11]. This multidimensional shaping of overall sensory style is also characteristic of Feng-flavor Baijiu. Originating in Shaanxi Province, Feng-flavor Baijiu is recognized as one of China’s four traditional famous Baijiu types [12] and is characterized by an “elegant and mellow aroma, sweet and moist yet brisk taste, harmonious flavor, and a clean, prolonged finish” [13,14]. Because its overall sensory quality is jointly determined by multiple attributes, it cannot be accurately characterized using a single indicator. Nevertheless, conventional sensory evaluation generally applies a uniform scale to assess individual attributes at the same hierarchical level, often overlooking differences in consumers’ attention to and decision weights assigned to these attributes. Some attributes may serve as core cues for style recognition and product acceptance, whereas others may play primarily supportive or coordinating roles. Therefore, determining the relative contributions of different sensory dimensions and specific attributes to consumers’ overall evaluations is essential for understanding the sensory quality and market-acceptance mechanisms of Feng-flavor Baijiu. To standardize the description and differentiation of complex sensory characteristics, existing studies commonly employ professional sensory evaluation systems based primarily on trained assessors. Although professional sensory evaluation offers high standardization and discriminatory capability [15], its evaluation criteria may differ from the actual perceptions and choices of ordinary consumers [16] and may therefore fail to fully reflect consumers’ genuine attention to different sensory attributes. Consequently, how consumers weigh the relative importance of multidimensional sensory attributes remains to be systematically elucidated.

Consumer evaluation of foods and beverages is inherently a process of integrating multiple attributes. When evaluating the same product, consumers selectively weigh sensory cues such as aroma, taste, and drinking perception, and the relative importance of these cues may be influenced by sensory sensitivity, consumption experience, product familiarity, and product expectations. Consequently, different consumer groups may develop distinct sensory priority structures. Among demographic factors, age has clear theoretical and practical relevance. On the one hand, olfactory and gustatory sensitivity, as well as the perception of and tolerance to oral stimulation, may differ across age groups [17]. On the other hand, consumers of different ages may also differ in drinking experience, consumption occasions, product exposure, and knowledge of Baijiu styles [18]. Previous studies have further suggested that consumers of different ages may exhibit different acceptance patterns for sweetness, sourness, aroma intensity, and mouthfeel characteristics [19]. However, little is known about how consumers of different age groups allocate evaluative weight across multiple sensory cues or whether their sensory decision structures differ systematically. Meanwhile, younger consumers are becoming increasingly important for Baijiu market segmentation and product innovation, whereas middle-aged and older consumers often have richer drinking experience and more established product knowledge. Therefore, clarifying age-related differences in sensory priorities is practically relevant for understanding consumer heterogeneity and supporting age-oriented market segmentation and product positioning. Accordingly, age was not regarded in this study as a causal determinant of specific sensory preferences, but rather as an important stratification variable associated with sensory capability, consumption experience, and product knowledge, allowing group-level heterogeneity in sensory decision structures to be examined.

Existing consumer sensory research on alcoholic beverages has extensively covered wine, beer, and no- or low-alcohol beverages. Previous studies have shown that product sensory characteristics, as well as consumer age and sex, can influence preference and emotional responses [19]. Surveys of Chinese wine consumers have further revealed regional differences in preferences for sweetness, aroma types, and mouthfeel, and corresponding preference-prediction models have been developed [20]. Consumer segmentation and multivariate analysis have also increasingly been applied to characterize heterogeneity in Baijiu consumption needs [21]. In comparison, consumer-oriented sensory research on Chinese Baijiu remains relatively limited. Existing studies on Feng-flavor Baijiu have mainly focused on sensory lexicons, typical style characteristics, flavor evolution during storage, and sensory–chemical relationships. For example, Ren et al. developed a flavor wheel and sensory lexicon based on 35 Feng-flavor Baijiu samples [22]. However, how sensory attributes at different hierarchical levels jointly shape consumers’ overall sensory priority structure, and how this structure varies across age groups, remain insufficiently understood.

Conventional mean comparisons, univariate tests, and simple rankings are limited in their ability to simultaneously characterize the hierarchical relationships, nonlinear associations, and complex interactions among sensory attributes. To address this limitation, the present study integrated hierarchical weighting analysis, machine learning, and SHAP. Hierarchical weighting analysis was used to quantify the relative importance of different sensory dimensions and specific attributes [23] and to construct a proportional sensory flavor wheel representing consumers’ overall multilevel sensory priority structure. Machine learning models, combined with cross-validation and feature-importance evaluation [23,24,25,26], were used to identify nonlinear combinations of sensory attributes associated with age-group classification. SHAP was then applied to further interpret the direction and relative magnitude of each attribute’s contribution to age-group classification. This integrated framework was designed to address the current lack of systematic understanding of the multilevel sensory priority structure of Feng-flavor Baijiu and its group-level variation from two complementary perspectives: shared consumer sensory priorities and age-related heterogeneity.

Accordingly, the present study was guided by two research questions: (RQ1) the characterization of the shared hierarchical structure of consumers’ stated sensory priorities for Feng-flavor Baijiu and (RQ2) the evaluation of age-group-associated information in sensory-weight configurations and the attributes contributing most strongly to group discrimination. To address these questions, this study aimed to: (1) establish a hierarchical consumer sensory evaluation framework and quantify the shared sensory-priority structure of the overall consumer sample; (2) use age as a predefined grouping variable to evaluate multivariate patterns in respondent-level sensory weights across age groups; and (3) integrate SHAP analysis with multiplicity-adjusted between-group comparisons to identify key age-discriminatory attributes and characterize their variation patterns. By distinguishing shared sensory priorities from age-group-related heterogeneity, this study sought to extend consumer research on Feng-flavor Baijiu from the perspective of relative sensory decision weights and provide a quantitative basis for subsequent consumer segmentation and product-based validation.

## 2. Materials and Methods

### 2.1. Participants

The questionnaire was administered using Questionnaire Star (Enterprise Standard Edition; Changsha Ranxing Information Technology Co., Ltd., Changsha, China). Participants were recruited nationwide through convenience sampling, primarily via online social media platforms such as WeChat. All respondents participated voluntarily after providing informed consent. Before the formal survey, a pilot test involving 50 participants was conducted to evaluate the clarity and comprehensibility of the questionnaire items. In addition, five internal experts reviewed the questionnaire content, and their feedback was used to refine the structure and wording of the final questionnaire. Data were collected from February to July 2026, yielding 1523 completed questionnaires. Sequential data screening was then performed according to the inclusion criteria of this study: age ≥ 18 years, previous experience of consuming Baijiu, and completion of the sensory-attribute weighting section in accordance with the required fixed-sum allocation. First, 28 questionnaires from respondents who did not meet the age criterion were excluded, leaving 1495 questionnaires. Second, 59 questionnaires from respondents without previous Baijiu consumption experience were excluded, leaving 1436 questionnaires. Finally, 79 questionnaires that did not satisfy the fixed-sum allocation requirements were excluded. Consequently, 1357 valid questionnaires were retained for subsequent analysis.

This study employed non-probability convenience sampling. Therefore, although the respondents were drawn from a broad geographic range, the sample should not be regarded as a probability-based representation of the overall population of Feng-flavor Baijiu consumers in China. In particular, recruitment through online social media platforms may have resulted in differential participation probabilities across age and other demographic groups. Accordingly, the external generalizability of the findings should be interpreted in light of the demographic composition of the sample.

### 2.2. Development of the Sensory Attribute System

For Feng-flavor Baijiu, a candidate sensory descriptor pool was first established based on GB/T 33405–2016 [27], ISO 13299:2016 [28], and sensory descriptors obtained from previous professional sensory evaluations. The candidate descriptors were then subjected to terminology standardization and screening for consumer applicability through the following steps: (1) descriptors that were ambiguous, difficult to perceive independently, or clearly beyond the comprehension of general consumers were removed; (2) synonymous and highly similar descriptors were merged, with expressions that were semantically clearer and easier for consumers to understand being retained; and (3) descriptors were prioritized according to their frequency of occurrence. Because some characteristic attributes of Feng-flavor Baijiu may occur at relatively low frequencies while still having substantial value for style representation, no single frequency threshold was used as the sole inclusion criterion. Instead, descriptor frequency, consumer comprehensibility, and representativeness of the Feng-flavor style were jointly considered during attribute selection.

The preliminary set of descriptors was then independently reviewed by five professionals with experience in Baijiu sensory evaluation, with particular attention to sensory distinctiveness, semantic independence, consumer comprehensibility, and representativeness of the Feng-flavor style. For descriptors for which opinions differed, the degree of definitional overlap and their value for style characterization were discussed collectively, and decisions to retain, merge, or remove descriptors were made after consensus was reached. Some low-frequency descriptors considered representative of the characteristic processing or sensory style of Feng-flavor Baijiu were retained. Ultimately, a consumer sensory attribute system comprising three primary dimensions—aroma, taste, and drinking perception—and 24 secondary attributes was established (Table 1).

To reduce potential misunderstanding caused by professional sensory terminology, each attribute was explained in language closely aligned with consumers’ everyday sensory experiences. A small-scale pilot test was conducted before the formal survey. Based on participants’ feedback regarding item clarity, attribute meanings, and response difficulty, the attribute definitions, questionnaire wording, and item order were revised to improve the comprehensibility and content validity of the survey instrument.

### 2.3. Questionnaire Design and Data Collection

The questionnaire consisted of two sections: respondent characteristics and fixed-sum weighting. The first section collected information on sex, age, Baijiu drinking frequency, familiarity with Feng-flavor Baijiu, and whether respondents had experience in industries related to Baijiu, food, sensory evaluation, catering, or alcoholic beverage sales. It should be noted that “drinking frequency” in this study referred specifically to Baijiu drinking frequency. According to the original questionnaire, respondents were classified into six frequency categories: 1–2 times per year, 3–6 times per year, 1–2 times per month, once per week, 2–3 times per week, and ≥4 times per week. Baijiu drinking frequency was used to characterize consumers’ general Baijiu consumption habits, whereas familiarity with Feng-flavor Baijiu was used to reflect product-specific knowledge and consumption experience; thus, the two variables represented different aspects of consumer background. These background variables were used primarily to characterize the study sample. Given that the main analytical objective of this study was to investigate shared sensory priorities and age-related heterogeneity, age was predefined as the primary grouping variable. Baijiu drinking frequency, familiarity with Feng-flavor Baijiu, and relevant industry experience were not included as input variables in the age-classification models, thereby avoiding conflating consumer background characteristics with the sensory-attribute weights used for age-group discrimination.

In the fixed-sum weighting section, respondents were required to allocate 100 points among sensory attributes within the same hierarchical level, with the total score across all attributes summing to 100. This fixed-sum constraint required respondents to make explicit trade-offs among attributes, thereby reflecting the relative importance assigned to each attribute in the overall evaluation of the product. To reduce the cognitive burden associated with simultaneously comparing a large number of attributes, the 100 points were allocated separately among the primary dimensions and among the secondary attributes within each primary dimension.

### 2.4. Development of the Weighting Analysis Method

To quantify consumers’ relative attention to sensory indicators at different hierarchical levels, a fixed-sum allocation method was used to calculate the weights of the primary dimensions, the local weights of the secondary attributes, and their global weights. Under a fixed total-score constraint, respondents were required to make explicit trade-offs among different indicators, thereby enabling the relative importance assigned to each attribute to be quantified [29,30]. In addition, following Saaty’s principle of hierarchical priority synthesis, the weights of the higher-level dimensions were integrated with the local weights of their corresponding lower-level attributes [31]. This approach enabled standardized comparisons among individual attributes while preserving the hierarchical relationships within the sensory evaluation system.

Specifically, each respondent first allocated 100 points among the three primary dimensions of aroma, taste, and drinking perception and subsequently allocated 100 points among the secondary attributes within each primary dimension. The calculations of the primary-dimension weights, secondary-attribute local weights, and global weights are presented in Table 2.

The overall local weight $\left(L_{gj}\right)$ represents the relative importance of a secondary attribute within its corresponding primary dimension and is therefore suitable for comparisons among attributes within the same dimension. The overall global weight $\left(G_{gj}\right)$ represents the relative contribution of a secondary attribute to the complete consumer sensory evaluation system, allowing comparisons among all 24 secondary attributes across dimensions and providing the quantitative basis for constructing the proportional sensory flavor wheel.

Bootstrap resampling was used to evaluate the stability of the weight estimates. In each iteration, a bootstrap sample of the same size as the original valid sample was drawn with replacement, and the primary-dimension weights, secondary-attribute local weights, and global weights were recalculated. This procedure was repeated 1500 times, and the 2.5th and 97.5th percentiles of the bootstrap distributions were used as the lower and upper limits of the 95% confidence intervals, respectively. The dimensions and attributes were ranked in descending order according to their overall mean weights.

### 2.5. Development, Evaluation, and Interpretation of Age-Group Classification Models

The respondent-level global weights of the 24 secondary sensory attributes were used as predictors in multiclass models for three predefined age groups: 18–30 years (n = 668), 31–45 years (n = 628), and ≥46 years (n = 61). Stratified random sampling test_size = 0.30, stratify = labels, and random_state = 42) produced a development set (n = 949; 467, 439, and 43 respondents, respectively) and a held-out test set (n = 408; 201, 189, and 18 respondents, respectively). The test set remained isolated during preprocessing, model selection, and hyperparameter optimization.

All preprocessing was implemented within an imbalanced-learn pipeline and refitted in each training fold. Features were standardized, screened using L1-penalized multinomial logistic regression, and balanced using SMOTE only within the training data. The selected regularization value (C = 0.10) retained all 24 attributes. Eleven algorithms—Gaussian Naïve Bayes, LightGBM, XGBoost, support vector machine, k-nearest neighbors, Extra Trees, CatBoost, gradient boosting decision tree, AdaBoost, Random Forest, and Decision Tree—were evaluated.

Algorithm comparison and hyperparameter optimization were performed in the development set using stratified nested cross-validation with five outer and five inner folds. RandomizedSearchCV evaluated up to 50 parameter combinations per algorithm, and macro-F1 was the prespecified model-selection metric. XGBoost achieved the highest outer-fold macro-F1 and was selected before the held-out test set was accessed. The complete search spaces and final pipeline settings are provided in Supplementary Table S1.

After the pipeline and hyperparameters had been fixed, XGBoost was refitted on the complete development set and evaluated once on the held-out test set. Accuracy; weighted, macro-averaged, and class-specific precision, recall, and F1-score; balanced accuracy; one-vs-rest AUC; and the confusion matrix were calculated.

TreeExplainer was then applied to the fitted XGBoost model using the held-out observations. Global importance was calculated for each attribute as the mean absolute SHAP value averaged across all observations and three class-specific outputs; class-specific absolute and signed SHAP values were also examined. Signed values were interpreted only for the specified class output, and SHAP values were treated as predictive contributions rather than measures of preference, statistical significance, or causality.

Analyses were performed in Python 3.9 using NumPy 1.26.4, pandas 2.2.2, SciPy 1.13.1, scikit-learn 1.3.2, imbalanced-learn 0.11.0, XGBoost 2.0.3, LightGBM 4.3.0, CatBoost 1.2.5, and SHAP 0.45.1.

### 2.6. Statistical Analysis

Cronbach’s α was used to assess the internal consistency of the questionnaire items. The Kaiser–Meyer–Olkin (KMO) test and Bartlett’s test of sphericity were used to evaluate sampling adequacy and the suitability of the data for factor analysis. Consumer sensory-attribute weights are presented as means with 95% confidence intervals, which were estimated using 1500 bootstrap resamples. For the key age-group classification attributes identified by machine learning and SHAP, differences in global weights among the 18–30-year, 31–45-year, and ≥46-year groups were assessed using the Kruskal–Wallis test. To control the risk of false-positive findings arising from multiple testing, *p* values were adjusted using the Benjamini–Hochberg procedure. Unless otherwise specified, a two-sided *p* < 0.05 was considered statistically significant; for multiple comparisons, an adjusted *p* < 0.05 was used as the significance criterion.

The questionnaire was administered online and data were collected using Questionnaire Star, Enterprise Standard Edition (Changsha Ranxing Information Technology Co., Ltd., Changsha, China). Consumer sensory-attribute weights were calculated using Microsoft Excel 2019 (Version 16.0). All figures were generated using Origin 2024 and the ChiPlot online platform.

## 3. Results

### 3.1. Participant Characteristics

After sequential screening, 1357 valid questionnaires were retained. The internal-consistency characteristics and correlation structure of the questionnaire data were subsequently examined. The overall Cronbach’s α was 0.81, exceeding the commonly accepted threshold of 0.70 and indicating acceptable internal consistency. However, because the questionnaire employed a hierarchical fixed-sum allocation format, Cronbach’s α was treated only as an auxiliary indicator and not as evidence of unidimensionality or complete psychometric reliability. The Kaiser–Meyer–Olkin (KMO) value was 0.985, and Bartlett’s test of sphericity was significant (χ^2^(276) = 28,530.06, *p* < 0.001). These results indicated that the inter-attribute correlation matrix had adequate sampling characteristics and was suitable for exploratory factor analysis. Nevertheless, the KMO measure and Bartlett’s test were interpreted as indicators of correlation structure and factorability rather than as direct evidence of construct validity. The appropriateness of the questionnaire content was additionally supported by relevant sensory standards, expert review, and consumer pilot testing, while the sampling stability and uncertainty of the estimated sensory weights were evaluated using 1500 respondent-level bootstrap resamples.

**Figure 1:**
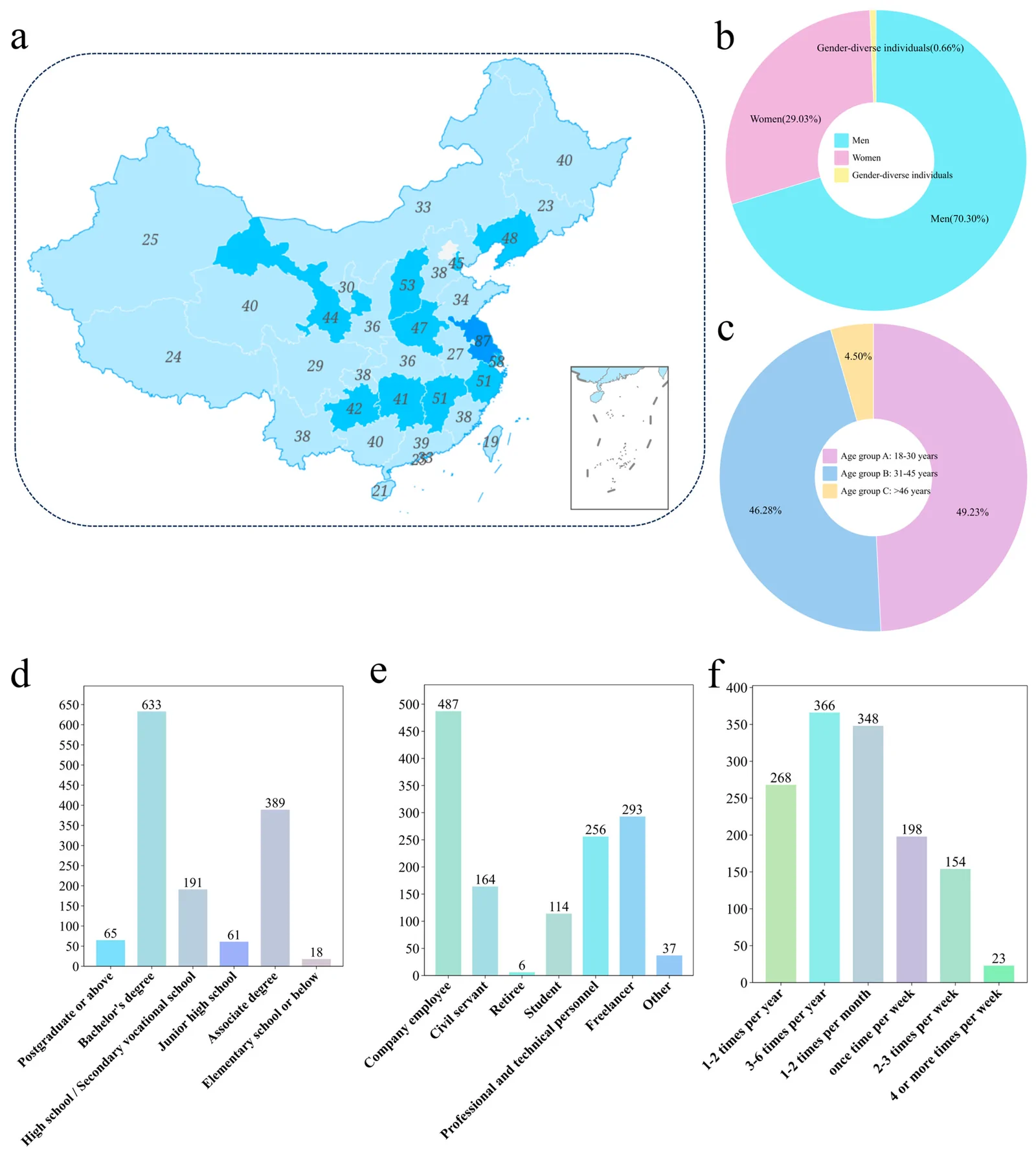
Demographic and behavioral characteristics of the respondents. (**a**) Geographic distribution; (**b**) gender distribution; (**c**) age distribution; (**d**) educational attainment; (**e**) occupational distribution; and (**f**) Baijiu drinking frequency. Data in (**b**,**c**) were rounded to two decimal places.

As shown in Figure 1a, respondents were recruited from all 31 provincial-level administrative regions of mainland China, as well as Hong Kong, Macao, and Taiwan, covering a total of 34 regions. The number of respondents from each region ranged from 19 to 87, with no marked concentration in a small number of areas. Jiangsu and Beijing contributed the largest numbers of respondents, with 87 (6.4%) and 84 (6.2%), respectively. Taiwan, Hainan, Jilin, and Tibet contributed relatively fewer respondents, accounting for 1.4%, 1.5%, 1.7%, and 1.8% of the sample, respectively. Each region accounted for less than 7% of the total sample, while Jiangsu and Beijing together accounted for 12.6%, indicating broad geographical dispersion.

Regarding gender distribution (Figure 1b), 954 respondents were male (70.3%), 394 were female (29.0%), and 9 identified as another gender (0.7%). Thus, the sample was predominantly male. Regarding age distribution (Figure 1c), 668 respondents were aged 18–30 years (49.2%), 628 were aged 31–45 years (46.3%), and 61 were aged 46 years or older (4.5%). Respondents aged 18–45 years accounted for 95.5% of the total sample, indicating that the study population was primarily composed of consumers within this age range.

Regarding educational attainment (Figure 1d), 633 respondents held a bachelor’s degree, accounting for 46.6% of the sample, while 65 respondents had postgraduate education or above, accounting for 4.8%. Together, respondents with a bachelor’s degree or higher represented 51.4% of the sample. A total of 389 respondents had completed junior college education (28.7%), 191 had completed senior high school or secondary vocational school (14.1%), 61 had completed junior high school (4.5%), and 18 had completed primary school or below (1.3%). Overall, 80.1% of respondents had received junior college education or above, indicating a relatively high educational level within the sample.

Regarding occupational distribution (Figure 1e), company employees constituted the largest group, with 487 respondents (35.9%), followed by freelancers, with 293 respondents (21.6%). Professional and technical personnel accounted for 256 respondents (18.9%), civil servants for 164 (12.1%), students for 114 (8.4%), respondents in other occupations for 37 (2.7%), and retirees for 6 (0.4%). Company employees, freelancers, and professional and technical personnel together accounted for 76.4% of all respondents.

Baijiu drinking frequency was relatively dispersed across categories (Figure 1f). The largest group comprised respondents who consumed Baijiu three to six times per year, with 366 individuals (27.0%), followed by those who drank once or twice per month (348; 25.6%) and once or twice per year (268; 19.7%). A total of 198 respondents drank once per week (14.6%), 154 drank two to three times per week (11.3%), and 23 drank four or more times per week (1.7%); overall, 72.3% consumed Baijiu less than once per week and 13.0% consumed it at least twice per week.

Regarding familiarity with Feng-flavor Baijiu, 34.27% of respondents reported limited familiarity but occasional consumption, 30.66% were relatively familiar and consumed it regularly, and 5.53% were highly familiar and frequently purchased or consumed it. Overall, 70.45% had consumed Feng-flavor Baijiu, 22.40% had heard of it but had never consumed it, and 7.15% had neither prior knowledge nor consumption experience. In addition, 47.75% had experience in industries related to Baijiu, food, sensory evaluation, catering, or alcoholic beverage sales, whereas 52.25% had no relevant professional background.

Overall, the sample consisted predominantly of male consumers aged 18–45 years, had a relatively high educational level, and mostly consumed Baijiu less than once per week; age was retained as the predefined grouping variable, while the other demographic characteristics were used descriptively.

### 3.2. Hierarchical Weight Analysis of Consumer Sensory Attributes

These results indicate that the study sample included Feng-flavor Baijiu consumers from diverse regions, age groups, and consumption backgrounds, thereby providing a data foundation for further examining overall sensory demands and age-related differences. Accordingly, hierarchical weighting analysis was first conducted across the full consumer sample to determine the relative importance of aroma, taste, drinking perception, and their corresponding secondary attributes within the overall evaluation system.

Based on 1357 valid questionnaires, this study established a consumer sensory evaluation system for Feng-flavor Baijiu comprising three primary dimensions and 24 secondary attributes and quantified the relative importance of each indicator using hierarchical weighting analysis. The results for the primary dimensions showed that drinking perception received the highest weight, at 35.23%, followed by aroma and taste at 33.54% and 31.22%, respectively (Table 3). The differences in weight among the three dimensions were relatively small.

The local weights of the secondary attributes showed that, within the aroma dimension, Jiuhai aroma received the highest weight (11.32%), followed by fruity aroma (9.75%), grain aroma (8.07%), and floral aroma (7.88%). The local weights of honey aroma, mellow aroma, sweet aroma, and light aroma were 7.58%, 6.93%, 6.90%, and 6.57%, respectively, while those of the remaining aroma attributes ranged from 5.49% to 6.52%. Within the taste dimension, sweetness had the highest local weight (33.77%), whereas sourness, saltiness, and bitterness accounted for 24.10%, 21.60%, and 20.53%, respectively. Within the drinking perception dimension, comfort received the highest local weight (20.59%), followed by purity (18.19%). The weights of harmony, smoothness, persistence, and fullness were 15.60%, 15.60%, 15.46%, and 14.56%, respectively.

**Figure 2:**
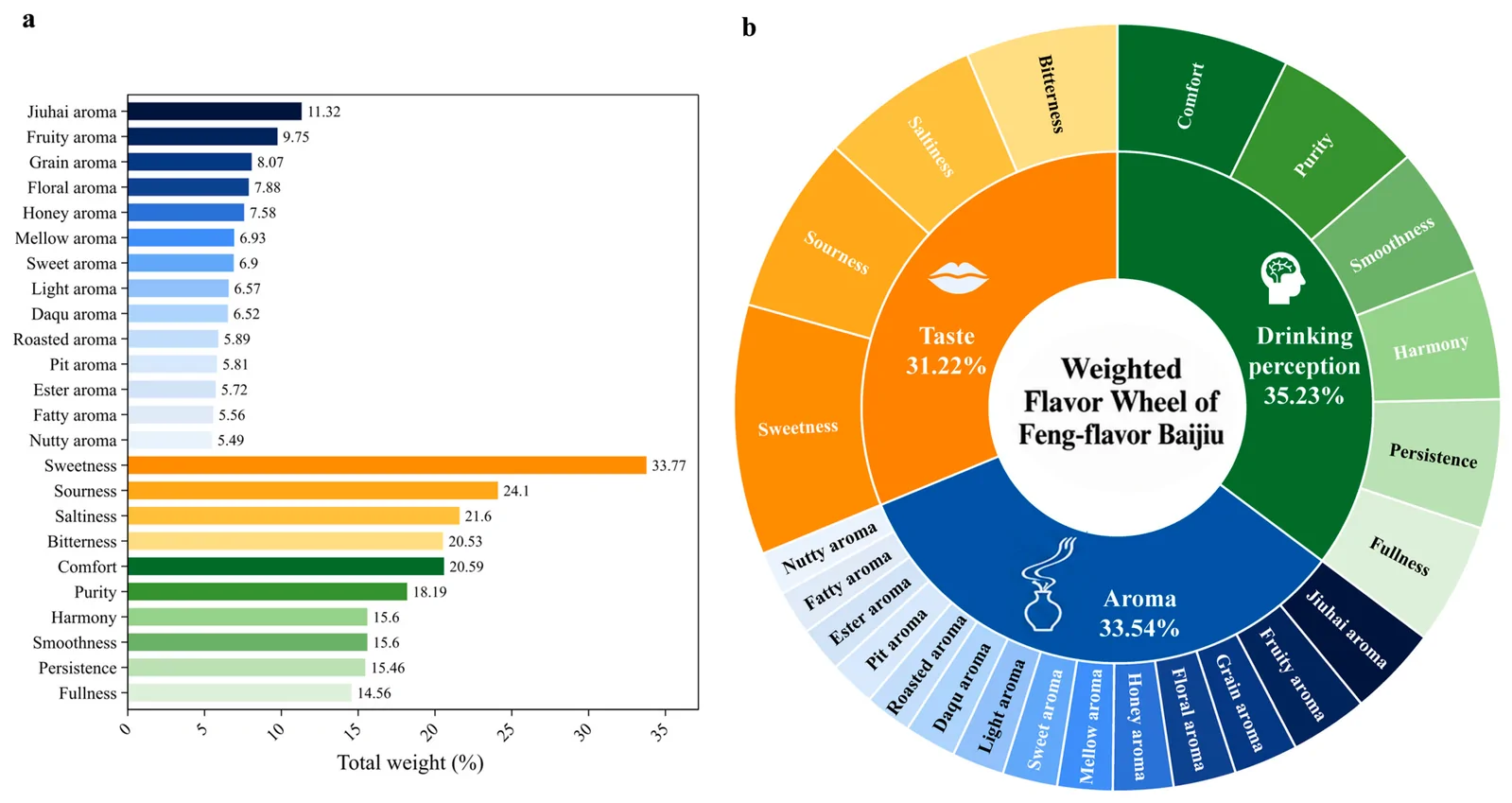
Sensory attribute weights and weighted flavor wheel for Feng-flavor Baijiu. (**a**) Ranking of the global weights of different sensory attributes; (**b**) weighted flavor wheel for Feng-flavor Baijiu. The data were rounded to two decimal places.

After integrating the primary-dimension weights with the local weights of the secondary attributes, sweetness had the highest global weight (10.23%). The global weights of comfort, sourness, saltiness, bitterness, and purity were 7.68%, 7.56%, 6.91%, 6.52%, and 6.13%, respectively (Figure 2a). Overall, taste- and drinking-perception-related attributes ranked relatively high in terms of global weight, whereas the global weights of aroma attributes were more widely distributed.

Based on these results, a hierarchical sensory attribute weight wheel was constructed (Figure 2b). The inner ring represents the overall weights of the three primary dimensions—aroma, taste, and drinking perception—whereas the outer ring displays the local weights of the secondary attributes within their corresponding dimensions. This visualization presents the hierarchical relationships and proportional distribution of the consumer sensory evaluation system for Feng-flavor Baijiu.

Further analysis showed that the sensory priorities of Feng-flavor Baijiu consumers were characterized by comparable importance across the primary dimensions but distinct patterns of weight distribution among the secondary attributes. The similar primary weights assigned to aroma, taste, and drinking perception indicate that consumers did not attribute product quality to any single sensory dimension; instead, they simultaneously considered aroma style, basic tastes, and the overall drinking experience. Nevertheless, the degree of weight concentration differed among dimensions. Attention within the taste dimension was concentrated primarily on sweetness, whereas comfort and purity received relatively greater attention within drinking perception. By contrast, attention within the aroma dimension was distributed across multiple attributes, including Jiuhai aroma, fruity aroma, grain aroma, floral aroma, and honey aroma, suggesting that consumers’ judgments of the aroma quality of Feng-flavor Baijiu were based on a more complex combination of sensory characteristics.

### 3.3. Development of Machine Learning Models for Consumer Age-Group Classification

**Figure 3:**
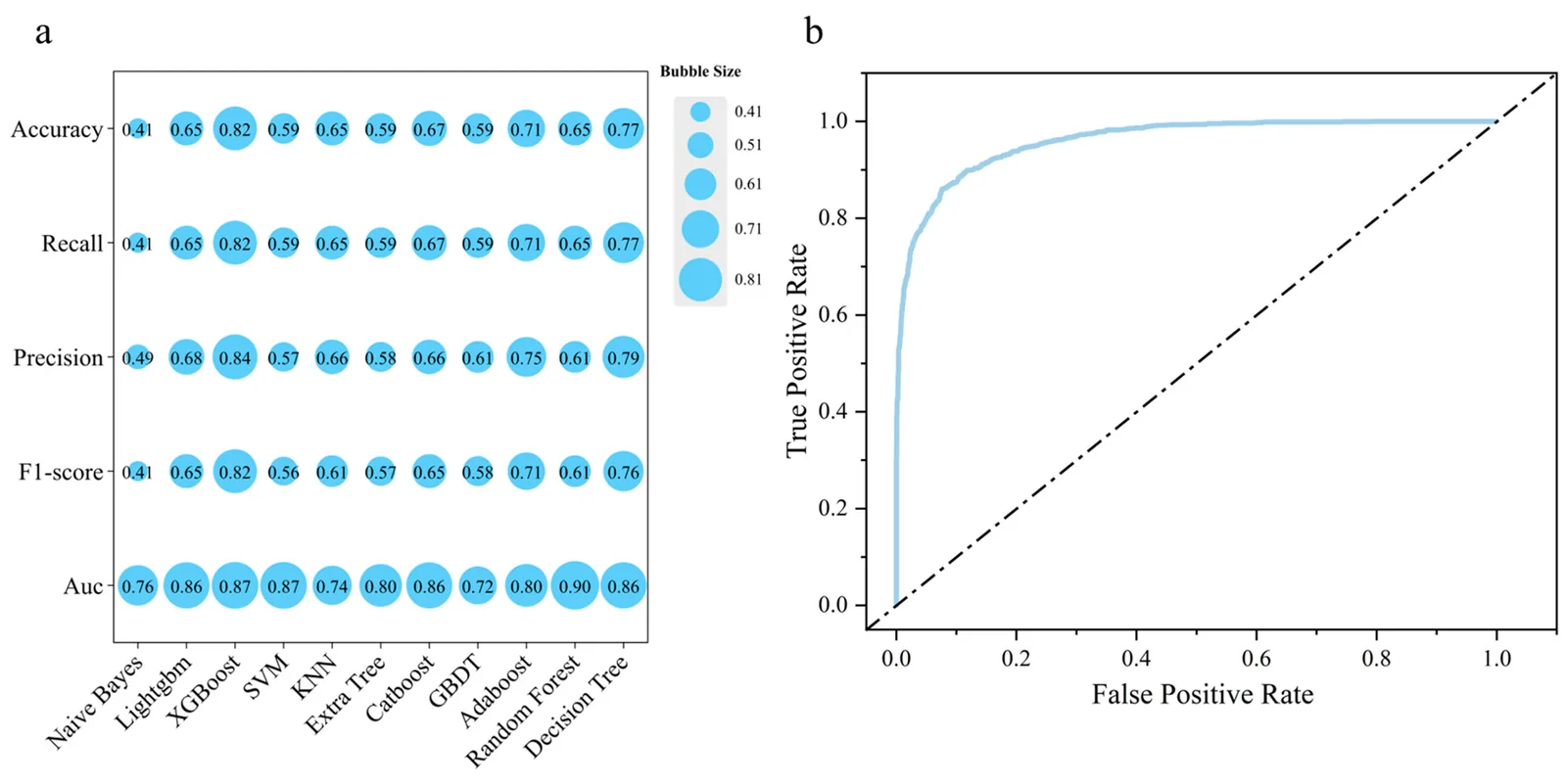
Predictive performance of the machine learning models evaluated using quantitative metrics. (**a**) Bubble plot comparing the performance of 11 models for age-group classification; (**b**) receiver operating characteristic (ROC) curves of the optimal model.

Eleven candidate algorithms were compared exclusively within the development set using outer-fold macro-F1 from the nested cross-validation procedure (Figure 3a; Supplementary Table S2). XGBoost achieved the highest mean outer-fold macro-F1 and was therefore selected before the held-out test set was accessed. After the complete pipeline and hyperparameters had been fixed, XGBoost was refitted on the full development set and evaluated once on the held-out test set. Its held-out performance is presented in Figure 3b and Supplementary Table S3.

On the held-out test set (n = 408), the final XGBoost model correctly classified 336 respondents (accuracy = 0.824). Weighted precision, recall, and F1-score were 0.836, 0.824, and 0.824, respectively; macro-precision, macro-recall, macro-F1, and balanced accuracy were 0.766, 0.760, 0.760, and 0.760, respectively. Class-specific F1-scores were 0.843, 0.824, and 0.611 for the 18–30-, 31–45-, and ≥46-year groups, respectively. The ≥46-year group contained only 18 observations in the held-out test set, of which 11 were correctly classified. Its lower F1-score and limited test-set size indicate greater uncertainty in the estimated predictive performance for this age group; therefore, the corresponding class-specific metrics should be interpreted cautiously rather than as stable estimates of performance in the broader population of older consumers.

The class-specific one-vs-rest AUC values were 0.88, 0.90, and 0.83 for the three age groups, yielding a macro-average AUC of 0.87 (Figure 3b). The confusion matrix showed 156, 169, and 11 correct predictions for the 18–30-, 31–45-, and ≥46-year groups, respectively (Supplementary Table S3).

### 3.4. Identification of Key Sensory Attributes Using SHAP

**Figure 4:**
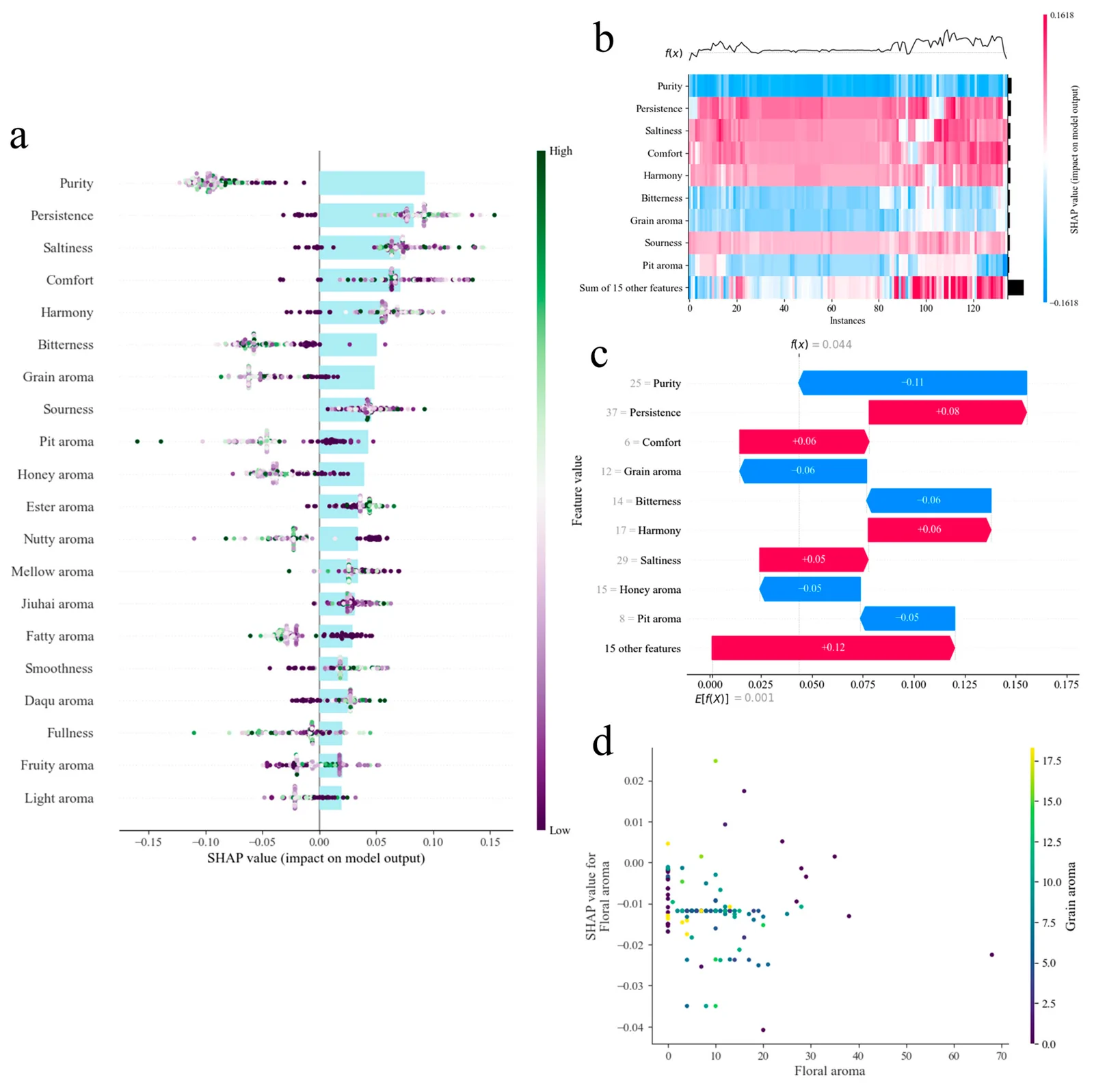
SHAP interpretation of the key sensory attributes associated with age-group classification among Baijiu consumers. (**a**,**b**) SHAP summary plots for the age-group classification results, simultaneously showing the global importance, direction of influence, and interindividual heterogeneity of each feature. (**c**) Local SHAP explanation for an individual prediction, illustrating the specific contribution of each feature to the model output for that sample. (**d**) SHAP dependence plot describing the relationship between the original feature value and its contribution to the model output.

SHAP analysis was conducted after the final XGBoost pipeline had been fixed and evaluated. Global mean absolute SHAP values were first calculated by averaging the absolute contributions across the 408 held-out test observations and the three class-specific outputs (Figure 4).

Purity showed the highest global mean absolute SHAP value (0.061), followed by persistence (0.057), saltiness (0.052), comfort (0.051), and harmony (0.048). Bitterness, grain aroma, sourness, pit aroma, and honey aroma formed a second group of relatively influential variables, with global mean absolute SHAP values of 0.046, 0.043, 0.042, 0.040, and 0.039, respectively. Sweetness had a comparatively low global SHAP importance of 0.022 despite having the highest overall global weight in the hierarchical weighting analysis (Figure 4a).

These results demonstrated that the overall sensory importance of an attribute was not equivalent to its discriminatory contribution to age-group classification. Sweetness and Jiuhai aroma had relatively high weights in the overall consumer hierarchy but provided comparatively limited additional information for distinguishing the three age groups. In contrast, purity, persistence, and saltiness contributed strongly to model classification even though their between-group mean differences were not necessarily large.

Class-specific SHAP analysis revealed that the relative contribution of the attributes differed among the three model outputs. For the 18–30-year output, saltiness had the highest class-specific mean absolute SHAP value (0.070), followed by purity (0.066), persistence (0.061), sourness (0.054), and harmony (0.052). Higher saltiness and sourness weights tended to increase the model output for the 18–30-year class, whereas lower values tended to decrease this class-specific output (Figure 4a).

For the 31–45-year output, purity (0.060), persistence (0.057), grain aroma (0.056), harmony (0.050), and saltiness (0.049) were the leading contributors. Higher grain-aroma weights tended to increase the model output for the 31–45-year class. However, the direction and magnitude of SHAP contributions varied substantially among individual observations, indicating that no single attribute independently determined assignment to this age group (Figure 4a).

For the ≥46-year output, comfort had the highest class-specific mean absolute SHAP value (0.066), followed by bitterness (0.063), purity (0.057), persistence (0.053), and honey aroma (0.050). Higher comfort, bitterness, and honey-aroma weights generally increased the model output for the ≥46-year class. Conversely, higher saltiness and sourness values generally made smaller or negative contributions to this class output (Figure 4a).

Purity and persistence contributed strongly to all three class-specific outputs. Their importance appeared to arise from differences in the multivariate configuration and distribution of weights rather than from large differences in their group means. Pit aroma showed a similar pattern: although its mean global weights were almost identical among the three age groups, it still contributed to classification when considered jointly with the other attributes.

The class-specific SHAP distributions also showed considerable interindividual heterogeneity. Attributes with high global importance did not exert effects of the same magnitude or direction for every observation. This variability further indicated that the XGBoost model classified age groups using combinations of aroma, taste, and drinking-perception weights rather than a single deterministic rule.

### 3.5. Age-Group Differences in Key Sensory Attributes

**Figure 5:**
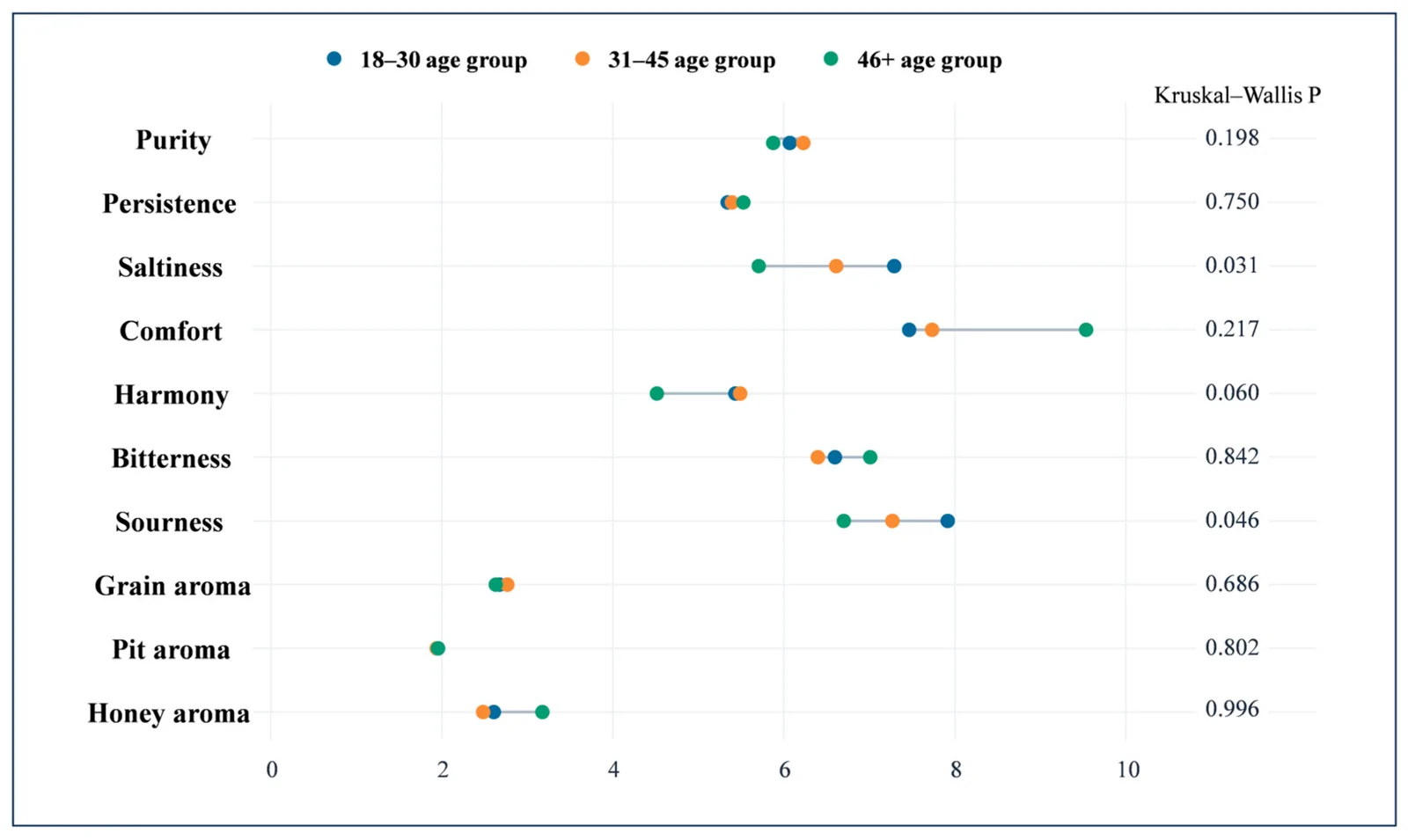
Differences in the sensory attribute scores for Feng-flavor Baijiu among consumer age groups. The colored points represent the mean global scores of consumers aged 18–30 years, 31–45 years, and ≥46 years, respectively. The Kruskal–Wallis test results are shown on the right; No comparison remained significant after BH correction.

SHAP analysis identified key sensory attributes with relatively high contributions to age-group classification. However, SHAP importance reflects only the relative discriminatory contribution of an attribute within a multivariate model and does not directly indicate the direction, magnitude, or statistical significance of between-group differences. Therefore, for the key age-discriminatory attributes identified by machine learning and SHAP, the Kruskal–Wallis test was further used to compare global scores among the three age groups, and the resulting *p* values were adjusted for multiple testing using the Benjamini–Hochberg procedure (Figure 5).

The global weights of saltiness and sourness both showed descriptive decreasing trends across the 18–30-year, 31–45-year, and ≥46-year groups. The mean global scores for saltiness were 7.29, 6.61, and 5.71, respectively, while those for sourness were 7.92, 7.27, and 6.70. In the unadjusted Kruskal–Wallis tests, the *p* values for these two attributes were 0.031 and 0.046, respectively. However, after Benjamini–Hochberg correction for multiple comparisons, the adjusted *p* values were 0.201 for both attributes. Therefore, these results indicate only an observed decreasing trend across age groups and do not support statistically significant age-group differences in either saltiness or sourness.

The mean global scores for harmony were 5.43 and 5.49 in the 18–30-year and 31–45-year groups, respectively, but decreased to 4.51 in the ≥46-year group, with a Kruskal–Wallis *p* value of 0.060. The mean scores for comfort were 7.47, 7.73, and 9.54 in the 18–30-year, 31–45-year, and ≥46-year groups, respectively. Although the ≥46-year group had the highest mean score, the within-group variability was also relatively large.

The mean global scores for purity ranged from 5.88 to 6.23 across the three age groups, while those for persistence ranged from 5.34 to 5.53, indicating relatively small differences in group means. Bitterness and honey aroma showed relatively higher mean scores in the ≥46-year group, whereas grain aroma was relatively higher among consumers aged 31–45 years. The mean global scores for pit aroma were 1.96, 1.94, and 1.95 across the three age groups, respectively, showing virtually no difference in mean values among groups.

## 4. Discussion

### 4.1. Shared Sensory Demand Structure of Feng-Flavor Baijiu Consumers

The hierarchical weighting results indicated that consumers assigned broadly comparable importance to aroma, taste, and drinking perception, although drinking perception received a slightly higher weight than the other two primary dimensions. This pattern suggests that consumers’ stated evaluation of Feng-flavor Baijiu is organized as an integrated, multidimensional judgment rather than being dominated by a single sensory domain. When evaluating alcoholic beverages, consumers may consider not only aroma and taste but also oral stimulation, body, aftertaste, and post-consumption responses [32]. The present findings extend this perspective by showing how consumers distribute limited evaluative weight among these dimensions and their subordinate attributes. Accordingly, the principal contribution of the weighting analysis lies not in identifying one universally dominant attribute, but in revealing the structure of the trade-offs consumers make when considering multiple sensory cues simultaneously.

Within this shared framework, the three primary dimensions exhibited different internal patterns of weight allocation. Aroma was represented by a relatively distributed set of priorities, with Jiuhai aroma, fruity aroma, grain aroma, floral aroma, and honey aroma receiving comparatively high local weights. Jiuhai aroma is a representative characteristic of Feng-flavor Baijiu, while Jiuhai, honey, and floral notes have previously been included among the attributes used to describe its sensory quality [13,22]. The distribution observed here therefore suggests that consumers’ stated judgments of aroma involve both recognition of product-specific style cues and consideration of more broadly familiar and pleasant aroma characteristics. Rather than relying on a single odor descriptor, consumers may form an overall judgment of aroma quality from several complementary cues.

By contrast, taste and drinking perception were concentrated around fewer attributes: sweetness received the highest weight within taste, while comfort and purity were prominent within drinking perception. Sweetness perception in Baijiu may reflect sweet compounds, taste interactions, and overall body balance [33,34,35]. These weights identify prominent cognitive priorities but do not indicate preferred product intensities.

Interpretation of the secondary-attribute weights must account for the fixed-sum and hierarchical questionnaire structure. Aroma contained 14 secondary attributes, compared with four for taste and six for drinking perception; thus, its available weight was distributed among more items. The lower global weights of individual aroma attributes therefore do not indicate that aroma was less important overall, because its primary-dimension weight was comparable to those of taste and drinking perception.

Taken together, the results reveal two complementary characteristics of the shared sensory-priority structure. First, consumers distributed importance relatively evenly across aroma, taste, and drinking perception, supporting a multidimensional view of Feng-flavor Baijiu evaluation. Second, the organization of priorities within each dimension differed: aroma was represented by the combined contribution of multiple attributes, whereas taste and drinking perception were more strongly concentrated around sweetness, comfort, and purity. Hierarchical weighting therefore provides information that cannot be obtained from a simple ranking of all attributes at a single level. It distinguishes the importance of a sensory dimension from the allocation of importance among attributes within that dimension and thereby offers a more structured representation of consumers’ stated sensory decision weights. The shared sensory-priority structure may vary by sex, and future studies should further examine sex-related differences using balanced or stratified samples.

### 4.2. Age-Related Heterogeneity in Consumer Sensory Demand

The machine learning, SHAP, and group-comparison results jointly suggest that age-group heterogeneity was expressed through multivariate configurations of sensory-attribute weights rather than through a uniform change in any single attribute [36,37]. This distinction is central to interpreting the study. The hierarchical weighting analysis addressed which attributes received relatively high importance in the full sample; the machine learning models evaluated whether combinations of respondent-level weights contained information associated with age-group membership; SHAP quantified the contribution of individual attributes to the model’s class-specific predictions; and the Kruskal–Wallis tests with Benjamini–Hochberg correction evaluated statistical evidence for differences in the original weight distributions. These quantities answer different questions and should not be interpreted interchangeably.

At the taste level, saltiness and sourness showed descriptive decreases across the 18–30-, 31–45-, and ≥46-year groups and contributed to age-group classification, especially for the 18–30-year output. However, neither attribute remained statistically significant after Benjamini–Hochberg correction; these patterns therefore represent descriptive trends rather than confirmed age-group differences.

The drinking-perception attributes provided particularly important multivariate classification information. Purity and persistence had high global SHAP importance despite showing only small differences in group means, while comfort contributed strongly to the ≥46-year class output and had a higher descriptive mean in that group. This apparent contrast illustrates why SHAP importance cannot be equated with a univariate effect size. An attribute may contribute to classification because of its distribution, nonlinear relationship with other variables, or role within a multivariate configuration, even when its group means are similar. Consumer judgments of alcoholic beverages can integrate mouthfeel, body, aftertaste, and other components of the consumption experience [32,38]. The current results are consistent with the possibility that age groups differ in the relative configuration of these drinking-perception cues.

Comfort, bitterness, and honey aroma were relatively prominent in the ≥46-year group, whereas grain aroma was relatively prominent in the 31–45-year group. These patterns may provide hypotheses about variation in product exposure, drinking experience, and emphasis on characteristic aroma or post-consumption experience [39,40]. However, because the ≥46-year group included only 61 respondents, with 18 represented in the held-out test set, the corresponding patterns should be considered exploratory, and their stability and generalizability to older consumers remain limited.

Some attributes with relatively high overall weights contributed less to age-group classification. Sweetness and Jiuhai aroma were important within the shared hierarchy but had comparatively low SHAP importance. Conversely, purity and persistence contributed strongly to classification despite limited differences in their group means. This divergence provides substantive information: attributes that are broadly important across consumers need not be useful for distinguishing consumer groups, whereas discriminatory attributes need not be the highest-ranked priorities in the full population. The results therefore support a conceptual distinction between shared sensory priorities and group-discriminatory information. The former may identify dimensions that warrant attention across the consumer population, while the latter may identify candidate indicators of consumer heterogeneity.

From a theoretical perspective, the observed age-group heterogeneity is better described as a reconfiguration of relative sensory decision weights than as a fixed directional shift in preference. Consumers across age groups appeared to share the same broad evaluation architecture—aroma, taste, and drinking perception—but differed in how relative importance was distributed among specific cues. This interpretation is consistent with a multi-attribute decision perspective in which consumers combine several sources of sensory information when forming an overall judgment [37,38]. It also avoids implying that age independently causes a preference for a specific attribute.

## 5. Conclusions

This study established a hierarchical framework for characterizing the stated sensory priorities of Feng-flavor Baijiu consumers and their associations with age. Aroma, taste, and drinking perception received comparable primary weights, indicating a shared multidimensional evaluation structure. Aroma priorities were distributed across multiple cues, whereas taste and drinking perception were concentrated around sweetness, comfort, and purity.

Age-related differences were primarily reflected in multivariate weight configurations rather than consistent changes in individual attributes. Machine learning and SHAP identified combinations associated with age-group classification. Saltiness and sourness decreased descriptively with age but were not significant after multiple-testing correction. Comfort, bitterness, and honey aroma were relatively prominent in the ≥46-year group, whereas grain aroma was prominent in the 31–45-year group. Purity and persistence contributed strongly to classification despite small differences in group means, highlighting the distinction between predictive contribution and statistical group differences.

The study conceptualized consumer heterogeneity as a reconfiguration of relative sensory decision weights within a shared framework. By integrating hierarchical fixed-sum weighting, machine learning, SHAP, and multiplicity-adjusted comparisons, the approach distinguished shared sensory priorities from group-discriminatory information. These findings provide a quantitative basis for identifying candidate sensory attributes for consumer segmentation, targeted product evaluation, and the age-oriented development and communication of Feng-flavor Baijiu. The identified attributes require product-based validation and should not be interpreted as direct formulation targets or confirmed preference drivers. The study was limited by convenience sampling, demographic imbalance, descriptor-based responses, and its cross-sectional design.

## Figures and Tables

**Table 1:** Hierarchical Sensory Attributes and Their Definitions Used in the Consumer Questionnaire for Feng-Flavor Baijiu.

Primary Attribute	Definition	Secondary Attribute	Definition
Aroma	The olfactory sensations perceived through orthonasal smelling or during consumption, including characteristic notes such as grain, Daqu, fruit, pit, and aged aromas.	Jiuhai aroma	A composite waxy, woody, and aged aroma associated with the storage of Baijiu in traditional Jiuhai vessels lined with Vitex twigs.
		Fruity aroma	An aroma reminiscent of fruits such as apple, pear, pineapple, banana, and melon, including mixed floral–fruity notes.
		Grain aroma	An aroma reminiscent of cooked grains, derived from cereal materials such as sorghum, rice, and wheat after fermentation and distillation.
		Floral aroma	A delicate and fragrant aroma reminiscent of natural flowers such as rose, osmanthus, locust flower, and gardenia.
		Honey aroma	A sweet and mellow aroma reminiscent of natural honeys such as locust honey, jujube-flower honey, and citrus-blossom honey.
		Mellow aroma	The characteristic aroma associated with alcohol compounds in Baijiu.
		Sweet aroma	A warm and sweet aroma reminiscent of caramel, rock sugar, or preserved ripe fruits.
		Light aroma	An elegant, clean, and pure aroma reminiscent of fresh wheat bran, grass, or steamed grains.
		Daqu aroma	The characteristic aroma imparted by fermentation starters such as Daqu, Fuqu, or Xiaoqu during Baijiu fermentation.
		Roasted aroma	An aroma reminiscent of roasted or toasted cereal grains.
		Pit aroma	A composite earthy and aged aroma associated with prolonged fermentation in traditional earthen fermentation pits.
		Ester aroma	A fruity, sweet, and composite aroma associated with esters such as ethyl acetate and ethyl hexanoate.
		Fatty aroma	A rich and rounded aroma reminiscent of cooked fats, oils, or oil-bearing seeds.
		Nutty aroma	A rich and oily aroma reminiscent of roasted nuts such as almonds, walnuts, and hazelnuts.
Taste	The basic taste sensations perceived by the tongue after the Baijiu enters the mouth, primarily including sweetness, sourness, bitterness, and saltiness.	Sweetness	A sweet, mellow, and smooth taste reminiscent of sucrose, honey, or polyols.
		Sourness	A fresh and lively acidic sensation reminiscent of organic acids such as malic acid, lactic acid, or acetic acid.
		Saltiness	A mild saline and umami-like sensation associated with trace mineral salts or amino acid salts, contributing to the perceived body of the Baijiu.
		Bitterness	A mild bitterness reminiscent of coffee, cocoa, or natural plant materials, without pronounced astringency or a lingering coating sensation on the tongue.
Drinking perception	The integrated sensations experienced in the mouth and during swallowing, including smoothness, fullness, harmony, purity, and aftertaste, together with post-consumption physical comfort.	Comfort	The overall physical comfort experienced after consumption, reflected by limited dry mouth, head discomfort, hangover sensations, or next-day headache.
		Purity	A clean and refreshing sensation without undesirable or extraneous sensory notes.
		Harmony	The degree to which the various sensory sensations in the mouth are well integrated and mutually balanced.
		Smoothness	The degree of softness and smoothness perceived when the Baijiu first enters the mouth.
		Persistence	The length of time for which the aftertaste remains perceptible after swallowing.
		Fullness	The perceived richness, complexity, and abundance of sensations in the mouth.

**Table 2:** Equations Used to Calculate the Hierarchical Weights of Consumer Sensory Attributes.

Equation No.	Formula	Interpretation
(1)	$p_{ig}=\frac{x_{ig}}{100}$	The allocation score for the primary dimension was normalized to obtain the individual weight assigned by respondent i to primary dimension g.
(2)	$W_{g}=\frac{1}{n}\sum_{i=1}^{n}p_{ig}$	The individual weights of all valid respondents were averaged to obtain the overall weight of primary dimension g.
(3)	$q_{igj}=\frac{y_{igj}}{100}$	The allocation score for the secondary attribute was normalized to obtain the individual local weight assigned by respondent i to attribute j within primary dimension g.
(4)	$L_{gj}=\frac{1}{n}\sum_{i=1}^{n}q_{igj}$	The individual local weights of all valid respondents were averaged to obtain the overall local weight of attribute j within its corresponding primary dimension.
(5)	$G_{gj}=\frac{1}{n}\sum_{i=1}^{n}\left(p_{ig}q_{igj}\right)$	The primary-dimension weight and secondary-attribute local weight were first combined at the individual level and then averaged across all valid respondents to obtain the overall global weight of attribute j.

Note: (i) denotes the respondent, where (i=1, 2,…,n); (*n*) denotes the number of valid respondents; (g) denotes the primary sensory dimension, including aroma, taste, and drinking perception, where (g = 1, 2, 3); (j) denotes the (j)th secondary attribute within primary dimension (g); and $\left(m_{g}\right)$ denotes the number of secondary attributes included in primary dimension (g). Aroma, taste, and drinking perception comprised 14, 4, and 6 secondary attributes, respectively. $\left(x_{ig}\right)$ denotes the score allocated by respondent (i) to primary dimension (g), with the scores assigned to the three primary dimensions summing to 100 for each respondent. $\left(y_{igj}\right)$ denotes the score allocated by respondent (i) to secondary attribute (j), with the scores assigned to all secondary attributes within the same primary dimension summing to 100. $\left(p_{ig}\right)$ and $\left(W_{g}\right)$ denote the individual and overall weights of the primary dimension, respectively; ($q_{igj}$) and $\left(L_{gj}\right)$ denote the individual and overall local weights, respectively; and $\left(G_{gj}\right)$ denotes the overall global weight. After normalization, the overall weights of the three primary dimensions sum to 1, the overall local weights of all secondary attributes within each primary dimension sum to 1, and the overall global weights of all 24 secondary attributes sum to 1.

**Table 3:** Results of the hierarchical weighting analysis.

Primary Attribute	Weight (%)	Lower 95% CI	Upper 95% CI	Secondary Attribute	Weight (%)	Lower 95% CI	Upper 95% CI
Aroma	33.54	32.77	34.36	Jiuhai aroma	11.32	10.59	12.07
				Fruity aroma	9.75	9.20	10.36
				Grain aroma	8.07	7.68	8.46
				Floral aroma	7.88	7.54	8.23
				Honey aroma	7.58	7.28	7.90
				Mellow aroma	6.93	6.68	7.19
				Sweet aroma	6.90	6.60	7.20
				Light aroma	6.57	6.32	6.84
				Daqu aroma	6.52	6.20	6.88
				Roasted aroma	5.89	5.62	6.19
				Pit aroma	5.81	5.54	6.11
				Ester aroma	5.72	5.51	5.97
				Fatty aroma	5.56	5.30	5.82
				Nutty aroma	5.49	5.26	5.75
Taste	31.22	30.62	31.89	Sweetness	33.77	32.57	34.88
				Sourness	24.10	23.29	24.97
				Saltiness	21.60	20.93	22.28
				Bitterness	20.53	19.89	21.18
Drinking perception	35.23	34.29	36.11	Comfort	20.59	19.84	21.40
				Purity	18.19	17.58	18.81
				Harmony	15.60	15.16	16.05
				Smoothness	15.60	15.09	16.11
				Persistence	15.46	15.06	15.88
				Fullness	14.56	14.12	15.01

## Data Availability

The original contributions presented in the study are included in the article/Supplementary Materials, further inquiries can be directed to the corresponding authors.

## Supplementary Materials

The following supporting information can be downloaded at: https://www.mdpi.com/article/10.3390/foods15162888/s1, Table S1: Preprocessing settings, hyperparameter search spaces, and final configuration of the XGBoost pipeline; Table S2: Quantitative performance metrics of the 11 candidate machine-learning models for age-group classification; Table S3: Class-Specific performance and confusion matrix of the final XGBoost model on the held-out test set.
